# Update on Ophthalmic Implications of Highly Pathogenic Avian Influenza A (H5N1) Virus

**DOI:** 10.3390/pathogens14090932

**Published:** 2025-09-16

**Authors:** Timothy Kaftan, Nam V. Nguyen, Jack Begley, Tolulope Fashina, Jessica Carag, Steven Yeh

**Affiliations:** 1 Truhlsen Eye Institute, Department of Ophthalmology, University of Nebraska Medical Center, Omaha, NE 68105, USA; 2 College of Medicine, University of Nebraska Medical Center, Omaha, NE 68198, USA; 3 Division of Infectious Diseases, School of Medicine, Emory University, Atlanta, GA 30308, USA; 4 Emory Eye Center, Department of Ophthalmology, Emory University, Atlanta, GA 30322, USA

**Keywords:** highly pathogenic avian influenza, H5N1, conjunctivitis, sialic acid residues

## Abstract

Introduction: Highly Pathogenic Avian Influenza (HPAI) A(H5N1) represents a growing public health challenge, given broadening zoonotic vectors, with a previously reported human mortality rate of roughly 50%. Late March 2024 marked the start of a new outbreak of HPAI A(H5N1) in the United States. While offering unique public health challenges, this outbreak also provides insight into clinical presentation and ocular involvement implications, transmission vectors, and the implementation of successful surveillance strategies. Objectives: This review seeks to highlight current and historical outbreak trends, transmission and ocular tropism significance, and strategies to mitigate viral spread. Methods: A comprehensive narrative literature review was completed using PubMed database as well as local, federal, and international public health press releases. Discussion: The 2024 US outbreak of H5N1 demonstrates the unique adaptability of the virus. Traditionally transmitted to humans via infected poultry, this outbreak marks the first confirmed case of dairy cow-transmitted human infection. Unlike many past H5N1outbreaks, the majority of patients in the current US outbreak have presented with conjunctivitis either alone or alongside systemic symptoms. This ocular-specific disease manifestation offers new clinical and screening implications. Awareness of ophthalmic involvement among physicians and public health organizations can help guide screening candidates and identify potential infections.

## 1. Introduction

Highly Pathogenic Avian Influenza (HPAI) A(H5N1), commonly referred to as H5N1, represents a growing public health challenge with ongoing zoonotic and human outbreaks. In late March 2024, H5N1 virus infection was identified in a dairy worker in Texas who developed bilateral conjunctivitis [1,2]. The majority of patients in the 2024 United States outbreak presented with conjunctivitis as either the sole presenting symptom or in association with systemic symptoms (Table 1).

Prior to 2024, ocular manifestations such as conjunctivitis rarely had been reported with H5N1 virus infection [3]. The recent findings provide insight into the importance of ocular tissue tropism as a potential conduit for exposure, inoculation, and a potential nidus associated with systemic involvement. Education of physicians and the public on the potential for ocular involvement may help to guide patient screening and prevent further spread. Another major finding from the 2024 US outbreak is the reported human transmission events via dairy cow viral hosts—a previously unreported animal vector [4,5].

Globally, the incidence of human infection remains low; however, reports of H5N1 spread have emerged outside endemic Asian regions to countries including the US [1], Canada [6], Australia [7], and the United Kingdom [8,9]. The global nature of spread with continued detection of H5N1 amongst humans, and changes in patient presentation underscore the importance of public health surveillance and clinician understanding of disease phenotypes. This review summarizes the current literature regarding the 2024 US H5N1 outbreak, viral pathogenesis, ocular manifestations, and implications for ophthalmologists and public health organizations.

**Table 1:** CDC-confirmed US H5N1 human infection events and corresponding ocular findings by state.

State	Number Infected (*n* = 70)	Infection Source (*n*)	Non-Ocular Findings (*n*)	Ocular Findings (*n*)
California [10]	38	Dairy cows (36), undetermined (2)	Fever (11), muscle aches (13), sore throat (6), cough (6), shortness of breath (4), vomiting (2), diarrhea (2), fatigue (7)	Conjunctivitis (37)
Washington [11]	11	Poultry * (11)	“Mild symptoms” (11)	Conjunctivitis (unspecified)
Colorado [12,13,14,15]	11	Dairy cow (1), Poultry * (10)	None (1), Fever, Chills, Cough, Sore Throat/Rhinorrhea (4), “Mild illness” [13] (2), “Mild symptoms” [14] (3)	Conjunctivitis/eye tearing (5)
Michigan [5,16]	2	Dairy cows (2)	None (1), cough (1)	Conjunctivitis (1)
Texas [1,17]	1	Dairy Cows	None	Conjunctivitis (1)
Ohio [18]	1	Poultry *	severe respiratory symptoms requiring hospitalization	Unspecified
Missouri [19]	1	Undetermined	Severe symptoms requiring hospitalizations	Unspecified
Oregon [20]	1	Dairy cow	Unspecified	Unspecified
Nevada [18]	1	Dairy cow	None	Conjunctivitis (1)
Wyoming [18,21]	1	Poultry backyard flock	Severe respiratory symptoms requiring hospitalization	Unspecified
Iowa [22]	1	Poultry *	“Mild symptoms”	Unspecified
Louisiana [23]	1	Poultry backyard flock	Severe respiratory symptoms requiring hospitalization, death	Unspecified

CDC-confirmed US H5N1 human infection events and corresponding ocular findings by state. This outbreak showcases previously rare ocular involvement and new zoonotic vector: the dairy cow. * Poultry from commercial or culling operations. Abbreviation: CDC, Centers for Disease Control and Prevention.

## 2. History of Animal and Human Outbreaks and Public Health Significance

### 2.1. Animal Outbreaks

The H5N1 strain was first identified in animals in Scottish poultry in 1959 [24]. In 1996, H5N1 was identified in a goose farm within China [25]. The virus eventually spread from commercial poultry to wild bird populations in Asia. The adaptability of the virus facilitated its transcontinental spread through migratory bird populations, eventually becoming endemic within Europe [2]. In 2022, the European Food Safety Authority (EFSA) reported 2398 poultry farm outbreaks in the United Kingdom and European Union [26]. This resulted in the death or culling of 46 million birds [26]. The EFSA has also reported H5N1 among avian populations across 31 European countries between 7 December 2024, and 7 March 2025 [27]. Since 2022, 51 US states or territories have reported detection of H5N1 virus in wild birds. Outbreaks have been reported in 51 US states or territories among poultry or backyard bird flocks and in 17 states among dairy cows [28]. Continued viral gene rearrangement has contributed to transmission and outbreaks reported among non-avian animal species such as minks [29], dairy cows [30], cats [30] and over 48 mammalian species [31]. With the expansion outside of endemic geographic areas, the addition of novel animal hosts, and over 170 million affected poultry in the United States alone, continued public health vigilance is warranted [28].

### 2.2. Human Outbreaks 

The first human infection by the HPAI subtype H5 was identified in Hong Kong in 1997 [32]. The outbreak included 18 cases, 6 of which resulted in patient death [32,33]. Human-avian interaction through both rural, backyard poultry and live poultry markets, in combination with an expansive avian population, has promoted the recurrence of outbreaks in the years following its identification as a human pathogen [34]. In 2003, human infections were reported amongst a Hong Kong family visiting the Fujian province in China [33]. Subsequent human outbreaks continued in neighboring countries such as Cambodia and Vietnam, with nearly annual outbreaks reported in China [35].

The World Health Organization has reported 472 fatalities from 984 cases of H5N1 from 1997 to January 2024 from 24 countries [32,36]. Transmission from avian species to humans is thought to occur primarily from exposure to infected, sick, or dead poultry [31,37]. Recent outbreaks in 2023 and 2024 have maintained the status of H5N1 as an ongoing public health threat. In Cambodia, 10 cases were reported in 2024, two of which were fatal [38]. The first case in Vietnam since 2022 was reported in March 2024, which was also fatal [39]. As of 29 May 2025, 70 human cases of H5N1 have been identified in the US (Table 1) [28]. Of these cases, one has resulted in death [23,28]. Recent human infections outside of the US include the first Australian H5N1 report in March 2024 [7], which was imported from Kolkata, India. A human case in England was reported in late January 2025 after exposure to infected poultry [9]. A confirmed human case has also been reported in Mexico in April 2025 [40]. The confirmed death in the current U.S. outbreak, combined with high mortality rates in outbreaks historically, and recent spread beyond traditional endemic regions, raises ongoing public health concerns globally.

### 2.3. Virology and Human Pathogenesis

Influenza A species circulating in avian populations are classified as low or highly pathogenic based by their level of morbidity and mortality in poultry. These naming conventions do not necessarily correlate with the severity of disease resulting from human infections [41]. Similarly to other influenza A viruses, H5N1 possesses important, antigen-specific surface glycoproteins that allow entry and transduction of viral RNA into host cells. These surface proteins include hemagglutinin (HA), neuraminidase (NA), and nonstructural proteins (NS). HA binds to specific host membrane sialic acid residues, facilitating viral membrane fusion. NA is a cleavage protein enabling viral release from infected host cells [41]. NS proteins are responsible for immune cell evasion and cytokine dysregulation [37,42,43]. The presence multi-basic cleavage sites (MBCS) in HA is unique to HPAI [44]. MBCS represents an acquired nucleotide alteration that translates to the synthesis multiple basic amino acids at the proteolytic cleavage site. Proteolytic cleavage is necessary for the activation of HA [44]. The altered cleavage site facilities promiscuous proteolysis at multiple tissue types contributing to systemic spread beyond characteristic intestinal or respiratory involvement [44]. Furthermore, genetic mutations within the gene encoding HA have been shown to confer differing tissue-specific binding affinity in isolates transmitted via animal hosts [44,45,46].

**Figure 1:**
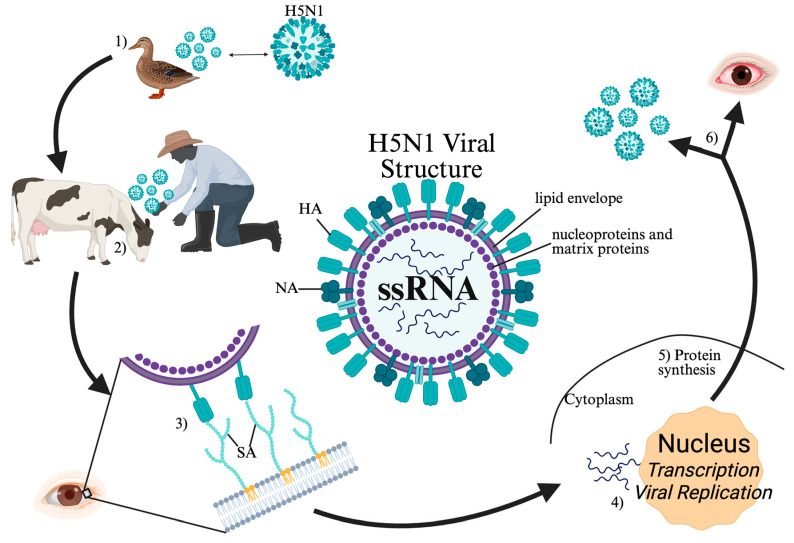
H5N1 structure and pathogenesis. (1) Avian reservoir species transmit H5N1 to intermediary hosts. (2) Human interaction with intermediary hosts enables human exposure. (3) Viral HA binds to host mucosal epithelium SA. The virus is then endocytosed and enters host cytoplasm surrounded by an endosome. Within the endosome the virus undergoes confirmational changes facilitating release of viral genome into the cytoplasm. (4) Viral RNA and protein complexes are transported into the nucleus where viral RNA polymerase complex initiates mRNA transcription and replication of negative sense viral RNA. (5) Viral genomic material exits the host nucleus into the cytoplasm where mRNAs are translated into proteins. (6) After assembly of progeny virion proteins and genome, apical plasma membrane budding and SA cleavage by NA facilities new viral formation and dissemination contributing to clinical manifestations such as conjunctivitis. Abbreviations: HA: hemagglutinin, NA: neuraminidase, ssRNA: single stranded RNA, SA: sialic acid.

H5N1 viral replication is thought to contribute to cell death by either direct cytolytic effects or via induction of an apoptotic pathway [37]. Viral replication occurs shortly after host inoculation, with measurable amounts of viral genes found one day after infection [37]. The site of infection is heavily influenced by sialic acid residues on host cellular membranes. H5N1 has a particular affinity for α-2,3-Gal residues which can be found in high density on human alveolar cells [47]. After insertion of viral machinery into host cells, host importin proteins transport protein-bound viral RNA and polymerase complexes into the nucleus. Within the nucleus, viral utilization of host proteins facilitates rapid transcription and viral replication [48]. H5N1 viral antigens are highly immunogenic. Hyperreactivity of local immune cells (particularly alveolar macrophages) results in upregulated cytokines such as tumor necrosis factor-alpha (TNF-α) and pro-apoptotic receptor ligands [37,43,47], leading to non-discriminant apoptotic activation. If viral clearance does not occur, this pattern of infection, replication, and tissue death is repeated leading to end-organ damage and the potential for further systemic spread (Figure 1).

## 3. Transmission from Animals to Humans

As a subtype of avian influenza, H5N1 spreads through its main host reservoir - wild waterfowl [31,41,49,50,51]. HPAI viruses replicate in multiple avian tissues with particularly high viral load in avian intestines [24,37,52]. These birds disseminate virus through saliva, feces and nasal secretions [24]. Viral spread to humans directly from wild aquatic birds is possible; however, more commonly, these reservoir birds transmit to an intermediary host—such as poultry—which then transmit H5N1 to humans through the handling of sick or dead intermediary host [53]. Outbreaks have been noted among poultry workers and, more recently, dairy cow workers in the US [29,49,54]. Its ability to overcome avian-specific tropism is conferred by its adaptive traits implicated in disease pathogenesis. In addition, the ability of the virus to proliferate in much lower temperatures of mammalian respiratory tracts highlights one of many troublesome genetic adaptations [24]. Moreover, the identification of mammalian adaptive genetic mutations in multiple organisms indicates mammal-to-mammal transmission capabilities [31,37].

To date, more than 48 mammalian species are known to have been infected with H5N1 [31]. Other potential avenues of animal-to-mammal transmission include the consumption of animal byproducts, as demonstrated by farm cat deaths following consumption of unpasteurized milk from infected dairy cows [55], and domestic and wild cat deaths after consumption of raw or untreated infected poultry products [56,57]. With recent US dairy farm worker infections and increasing documentation of infection in other domestic species, transmission to humans via infected mammals or their byproducts should be examined as a potential viral source. 

## 4. Surveillance in the United States

The CDC maintains its surveillance of HPAI in the US through a network of local and federal reporting systems. The CDC utilizes the One Health approach, whereby public health is optimized through strategic collaborations with diverse human, animal, and environmental health partners. The US Department of Agriculture communications have reported over 13,000 infected wild birds and 1700 poultry outbreaks affecting over 170 million poultry since 2022. Surveillance of dairy cattle infections has identified over 1000 affected herds as of May 2025 [28]. The success of animal surveillance allows for efficient determination of high-risk individuals. Utilization of local healthcare and emergency department case reporting, public health laboratory monitoring, clinical laboratory trends, and wastewater surveillance provides the framework for CDC infection identification efforts. Since February 2022, the department has monitored over 16,700 individuals with known exposure to infected animals and tested at least 880 individuals [28]. Through collaboration between local health departments, the CDC-led H5 surveillance program has successfully detected 64 human cases. Partnership among public health agencies and clinicians to identify high-risk populations has allowed for prompt US outbreak identification. 

## 5. Systemic Findings and Disease in Animals and Humans

### 5.1. Clinical Findings in Animals

The disease severity of H5N1 virus infection in birds ranges from subclinical infection to death, with the severity of disease often proportional to viral load [58]. In birds with severe disease, necrosis of neuronal, renal, cardiac, pancreatic, adrenal, and pulmonary tissue has been noted [52,58,59]. In chickens, necrotic nasal mucosa was reported 24 h following direct inoculation, demonstrating the potential for rapid progression of infection severity [58]. Similar multiorgan failure has been seen in other infected animals [29]. US dairy cows involved in the current outbreak have been reported to exhibit non-specific clinical findings with decreased lactation [30]. Milk samples can contain high levels of virus. Postmortem findings include mastitis and mild lymphocytic or neutrophilic hepatitis [30].

### 5.2. Clinical Findings in Humans

The clinical presentation from human infection also displays a wide range of severity. Symptomatic patients often present with headache, myalgia, and respiratory symptoms such as coughing or shortness of breath [24,50,60]. A growing number of recent cases also report ocular findings of conjunctivitis [1,4,61]. Classic respiratory manifestations can be attributed to viral propensity towards type II alveolar cells [50]. Progression of respiratory symptoms is often the cause of mortality as patients develop pneumonia, respiratory failure, and subsequent death [30,43,60,62]. Extrapulmonary manifestations have also been noted with infections identified in spleen, lymph node, bone marrow, brain, and liver tissue [37,50].

The association of ocular findings in individuals infected with H5N1 may be suggestive of viral tropism similar to that of respiratory tissue. This similarity may be explained by the presence of α2-3-linked and α2-6-linked sialic acid residues in both the corneal and conjunctival epithelium, as well as in the respiratory tract [60,63]. As mentioned previously, these sialic acid residues are integral to HA binding and subsequent cellular infection. Recent analysis of H5N1 clade 2.3.4.4b strains isolated from cattle showed altered HA residues with slightly increased binding affinity towards α2-6-linked sialic acid residues with retention of α2-3 sialylated glycan binding affinity as demonstrated in an in vitro model [45]. This clade has been implicated in the 2024 US outbreak, and its altered HA protein structure may play a role in its unique clinical features [45]. Additionally, H5N1 strains isolated from human conjunctival swabs were studied in human corneal constructs and ferret models [64]. These models demonstrated the capacity of H5N1 to infect and replicate in corneal constructs; however, viral titers were significantly lower compared to those observed with H7N7 and H1N1. The authors did not find features indicative strain-specific increased ocular tropism and call into question the role exposure route may have in contributing to ocular manifestation prevalence [64]. Further research is required to fully elucidate the degree to which H5N1 HA binding affinity contributes to human conjunctival cell entry. Clinically, infection of ocular surface cells manifests as conjunctivitis. Few ophthalmic findings besides conjunctivitis have been associated with H5N1 infection. Concomitant subconjunctival hemorrhage has been reported during the 2024 US outbreak [1].

## 6. Ophthalmic Implications and Evaluation Recommendations 

### 6.1. Risk Factor Assessment

When determining whether to pursue H5N1 evaluation, infection risk factors are important considerations. Exposure history may be instrumental in the identification of high-risk populations, while patient symptoms and disease findings may also be characteristic. 

Given that wild and domestic birds remain common hosts, a history of handling or consumption of sick or dead wild birds and poultry or exposure to wet poultry markets should be assessed when considering HPAI [54]. H5N1 has classically been transmitted to humans through interaction with poultry [59,65,66,67,68,69]. With the recent reports of dairy cattle-to-human transmission [1], symptomatic patients with exposure to dairy cows should be considered for evaluation. Given these exposures, dairy and poultry workers, butchers, meatpackers, and bird hunters remain relatively high-risk populations [1,30,70,71].

While ocular involvement was rarely documented previously, most human H5N1 cases identified in the U.S. in 2024 presented with conjunctivitis [4,10]. Thus, patients with conjunctivitis presenting with or without acute respiratory symptoms should be assessed for recent poultry or other animal exposures, including dairy cattle. 

### 6.2. Ophthalmic Implications 

Recent reports of conjunctivitis in dairy and poultry farm workers with H5N1 virus infections in the U.S. have heightened the importance of consideration of viral inoculation of the ocular surface. Ferret and guinea pig models support conjunctival infection secondary to deposition of virus in droplets or aerosols onto conjunctival tissues [72,73]. Direct inoculation of the eye by virus-contaminated gloves or hands/fingers of dairy workers has been hypothesized as a potential route of inoculation [1]. Direct inoculation is also supported by a dairy farm worker who developed conjunctivitis after being splashed in the face when milking a cow [4]. Cases of patients with conjunctivitis in the absence of systemic or respiratory symptoms support the eye as the site for primary infection [1,4,61]. Additionally, cases of H5-positive conjunctival swabs with negative nasopharyngeal swabs indicate the importance of ocular epithelial tropism in disease [74]. These findings may support the notion that levels of viral shedding vary across mucosal sites, potentially influenced by unique microenvironments of each location. Increased viral detection at the conjunctiva compared to respiratory mucosal membranes may be explained by the relatively exposed nature of anterior ocular tissue and its unique local immune response [75]. While not immune privileged, the conjunctiva possesses immune regulatory mechanisms that contribute to a degree of antigen tolerance [75]. These mechanisms include goblet cell secretion of TGF-β2, tolerogenic dendritic cell phenotype, and homeostatic lymphoid and myeloid immune cell populations as seen in mice models [75,76,77]. However, further research is required to determine the role–or lack thereof–these mechanisms may play in the injectivity of conjunctival tissue by H5N1.

In contrast, other human influenza viruses have historically been associated with ophthalmic involvement [60,62,68,78]. Ocular manifestations such as retinopathy, optic neuritis, and uveitis have been reported with H1N1 influenza [62]. Additionally, other HPAI H7 subtypes have long been associated with conjunctivitis in human infection [78]. An investigation into the 2003 H7N7 outbreak in the Netherlands showed 83 of 89 infected patients presented with conjunctivitis [68]. In 86 patients, infection was attributed to occupational exposure to infected poultry [68]. However, infection in the remaining 3 patients was attributed to human-to-human transmission from family members working with the diseased poultry flock [68]. Patients involved in human-to-human transmission were reported to have conjunctivitis [68,79]. While no H5N1 human-to-human transmission has been reported to date, the recent shift towards ocular tropism exemplifies viral adaptability, which may continue to expand the list of transmissible viral hosts. 

Further study of the Netherlands H7N7 outbreak expanding into 2004 found that 349 out of 453 individuals with health complaints presented with conjunctivitis [80]. Notably, H7 conjunctival swabs were positive in 6 patients without clinical ocular symptoms [80]. 

In 2013, an outbreak of H7N7 was identified in Italy involving 3 patients. These patients were exposed to the virus through culling of poultry suspected of H7N7 infection [81]. Two patients worked with the poultry without personal protective equipment (PPE) prior to H7N7 poultry outbreak discovery. All patients wore PPE during the culling process. The three patients presented with conjunctivitis without respiratory symptoms. Each patient’s conjunctival swabs were positive for the virus. They were isolated at home and symptoms resolved without antiviral treatment [81].

These human outbreaks highlight the importance of ocular tissue for HPAI viral infection (Table 2). Conjunctivitis occurs in about 80% of human H7 infections [60]. Typically these cases are self-limited, and no cases of permanent vision loss associated with H5 or H7 ocular infections were identified in this review. 

### 6.3. Personal Protective Equipment (PPE) Recommendations: Public Health Guidance from the CDC and the American Academy of Ophthalmology (AAO)

The CDC recommends the use of PPE when “working directly or closely with sick or dead animals, animal feces, litter, raw milk, and other materials that might have the virus [87].” Hand washing before and after PPE donning and doffing plays an important role in infection mitigation [71]. Recommended PPE for occupational exposure includes fluid-resistant coveralls, a respirator approved by The National Institute for Occupational Safety and Health (NIOSH) (e.g., fit-tested N95 filtering facepiece respirator), goggles or facemask, headcover, gloves and boots [87]. While working in PPE, it is important to avoid eating, drinking, smoking, and touching the eyes, mouth, or nose [87].

Postexposure prophylaxis (PEP) is recommended for those with high-risk exposure such as contact with known infected individuals or animals without recommended PPE [88]. H5N1 PEP consists of 5 days of oral oseltamivir (generic or brand-name Tamiflu [Genentech USA, Inc., San Francisco, CA, USA]) administered twice daily [88]. Individuals receiving PEP should also undergo influenza A(H5) testing when feasible [88]. 

The AAO also recommends that ophthalmologists maintain alertness for the possibility of H5N1 in patients with conjunctivitis and particularly, if risk factors exist including exposure to sick or dead birds, dairy cows or livestock [89].

In patients with conjunctivitis requiring testing, it is advised to perform one conjunctival swab and one nasopharyngeal swab. For those without conjunctivitis, one nasopharyngeal swab, one nasal swab, and one oropharyngeal swab are recommended [89]. Treatment with twice daily oral oseltamivir should not be delayed by pending lab results and should be given to all patients regardless of severity at the time of presentation [89]. Overall patient health should be assessed when considering hospitalization needs. Given the association between respiratory failure and increased mortality, patients with preexisting respiratory disease–such as chronic obstructive pulmonary disease–should be appropriately monitored with the involvement of a multidisciplinary care team. 

## 7. Discussion and Future Directions

Given the potential for inoculation of ocular surface cells by H5N1, protective ocular precautions are recommended for individuals working with animal products contaminated by virus or if there is potential for occupational exposure. 

Clinical recognition of conjunctivitis as an early disease manifestation can assist in the diagnosis with the potential for earlier treatment. Patients in the current US outbreak have responded to oral neuraminidase inhibitors–such as oseltamivir–which saw conjunctivitis and disease resolution [1,4]. Vaccines are currently under investigation and may play a role as a preventive measure in the future [90,91]. A Phase 2 clinical trial of the IVACFLU A/H5N1 vaccine showed an acceptable safety profile and immunogenicity [91]. Additionally, a phase 3 MF59-adjuvanted H5N1 vaccine showed protective anti-HA titers at 43 days; however, 6-month titers fell below target levels [92]. Continued public health surveillance, prompt clinical recognition and treatment, and vaccine strategies may also play a role in risk reduction for future H5N1 infection and spread. 

Furthermore, additional investigation into pathobiological mechanisms of H5N1 ocular infection is required for better understanding of the prevalence of ophthalmic involvement in the US outbreak. Future research in human ocular histopathology may elucidate valuable insight into H5N1 conjunctival cell entry.

## 8. Conclusions

The current zoonotic H5N1 outbreaks among poultry and dairy cattle in the US and resultant animal-to-human transmission present challenges given the scope of the outbreak and potential for expansion. The spread from Asian countries [93,94,95] to global prevalence [1,29,49,96] as well as novel mammalian hosts underscores the importance of continued surveillance and interdisciplinary, coordinated prevention efforts with One Health approaches. Given the cases of conjunctivitis associated with H5N1 and the potential that ocular involvement may be an early sign of infection, adherence to CDC and AAO guidelines is also paramount for the detection of disease and prevention of transmission.

## Figures and Tables

**Table 2:** Summary of published H7 human outbreaks and corresponding ocular manifestations.

Author (Year)	Country	H7 Subtype	Number of Cases	Ocular Manifestations (*n*)	Fatalities
Fouchier et al. [68] (2004); Du Ry van Beest Holle et al. [79] (2005)	Netherlands	H7N7	89	Conjunctivitis (83)	1
Koopmans et al. [80] (2004) *	Netherlands	H7N7	453 (symptomatic patients)	Conjunctivitis (349)	1
Tweed et al. [82] (2004)	Canada	H7N3	2	Conjunctivitis (2)	0
Belser et al. [66] (2013)	Mexico	H7N3	2	Conjunctivitis (2)	0
Li et al. [83] (2014)	China	H7N9	139	None Reported	47
Puzelli et al. [81] (2014)	Italy	H7N7	3	Conjunctivitis (3)	0
Wang et al. [84] (2017) **	China	H7N9	1220 (from 2013–2017)	None Reported	Not Reported (morality ∼39% [85])
Terebuh et al. [86] (2018)	United States	H7N2	1 (serology testing from 2002 exposure)	None Reported	0

Summary of published H7 human outbreaks and corresponding ocular manifestations. Conjunctivitis was the most common ocular manifestation with instances where it was the only presenting symptom. * Data includes previously published data by Fouchier et al. ** Data includes previously published data by Li et al.

## Abbreviations

The following abbreviations are used in this manuscript:

HPAI	highly pathogenic avian influenza
EFSA	European Food Safety Authority
HA	hemagglutinin
NA	neuraminidase
NS	nonstructural proteins
MBCS	multibasic cleavage sites
TNF-α	tumor necrosis factor-alpha
CDC	Centers for Disease Control and Prevention
NIOSH	The National Institute for Occupational Safety and Health
PPE	Personal Protective Equipment
AAO	American Academy of Ophthalmology
PEP	Postexposure prophylaxis
